# IL-32θ inhibits stemness and epithelial-mesenchymal transition of cancer stem cells *via* the STAT3 pathway in colon cancer

**DOI:** 10.18632/oncotarget.7007

**Published:** 2016-01-25

**Authors:** Yesol Bak, Taeho Kwon, In seon Bak, Jintae Hong, Dae-Yeul Yu, Do-Young Yoon

**Affiliations:** ^1^ Department of Bioscience and Biotechnology, Bio/Molecular Informatics Center, Konkuk University, Seoul, Korea; ^2^ Disease Model Research Laboratory, Aging Intervention Research Center, Development and Differentiation Research Center, Korea Research Institute of Bioscience and Biotechnology (KRIBB), Daejeon, Korea; ^3^ College of Pharmacy, Chungbuk National University, Cheongju, Chungbuk, Korea

**Keywords:** IL-32, cancer stem cells, stemness, EMT, colon cancer

## Abstract

Interleukin (IL)-32 is a well-known cytokine associated with inflammation, virus infections and cancer. IL-32θ is a newly identified isoform of IL-32, whose function has yet to be elucidated. In this study, we investigated IL-32θ function in colon cancer stem cells. Using samples from colon cancer patients, we found that the expression of IL-32θ mRNAs was significantly suppressed in tumor regions. We investigated the effects of IL-32θ on colon cancer. Ectopic expression of IL-32θ attenuated invasion, migration *in vitro* and *in vivo* tumorigenicity of colon cancer cells. IL-32θ inhibited epithelial-mesenchymal transition (EMT), resulting in the suppression of their migratory and invasive capabilities of HT29 colon cancer cells. In addition, IL-32θ altered various properties of CSCs, including sphere formation and expression of stemness related genes. IL-32θ directly bound to STAT3 and inhibited its nuclear translocation, leading to inhibited transcription of downstream factors, including Bmi1 and ZEB1. We showed that IL-32θ inhibited the STAT3-ZEB1 pathway and consequently inhibited key factors of stemness and EMT. Taken together, our findings reveal that IL-32θ can be a tumor suppressor, indicating that IL-32θ could possibly be used in therapies for colon cancer.

## INTRODUCTION

Colon cancer is the second most common cause of cancer-related deaths. Some cancer cells, such as cancer stem cells (CSCs) are resistant to current therapies [1]. CSCs were first identified in cases of acute myeloid leukemia [2] and were found to be a rare sub-population of cells with high tumor initiation capacity, within a bulk tumor mass. Jordan *et al*. reported that CSCs recapitulate the entire tumor population *in vitro* and *in vivo* [3]. There is evidence to suggest that CSCs are found in brain [4], breast [5], colon [1] and ovarian cancer [6]. CSCs are correlated with cancer progression, drug resistance and recurrence [7]. CSCs also possess the capacity for self-renewal, similar to that for undifferentiated hematopoietic stem cells [8] and express markers of stem cells, relying on similar pathways for their proliferation [4]. The self-renewal properties of CSCs promote tumorigenesis [9]. Despite the large amount of available data for various cancer therapies, relatively little information is available regarding CSC-targeted therapies [10]. Current cancer therapies only eliminate differentiated cancer cells; therefore, understanding and being able to control the functions of CSCs would be of immense clinical interest.

Constitutive activation of signal transducer and activator of transcription 3 (STAT3) contributes to the maintenance of colon cancer [11, 12] and colon cancer-initiating cells [13]. The STAT3 signaling pathway is a representative oncogenic pathway in cancer. Abnormalities in this pathway during colon cancer have been highlighted by several researchers [14, 15]. However, the mechanism underlying STAT3 inhibition in colon cancer is not clearly understood.

CSC fate is determinated by intrinsic and extrinsic pathways, including cytokine networks [7, 16]. It is possible that cytokines induce many of the properties of cancer cells and CSCs. IL-6 and IL-8 induce elevated levels of CSC self-renewal in breast cancer [17, 18], while IL-1β stimulates the stemness and invasiveness of CSCs in colon cancer [19]. Many researchers reported that IL-32 has regulatory effects on cancer. IL-32α suppresses colorectal cancer development [20], but is involved in hepatocellular carcinoma [21]. Also, IL-32β induces migration of breast cancer cells [22], whereas promotes cytotoxic lymphocyte activation and NF-κB, STAT3 inactivation [23]. Recently, our lab identified a new isoform of IL-32 which is called IL-32θ, but its role in the colon cancer is not entirely clear as yet. IL-32θ has sequence similarities with IL-32β, but lacks exon 6 [24]. We previously found that IL-32θ effectively inhibits STAT3 transcriptional activity [24]. In this study, we investigated the role of IL-32θ in colon cancer, with an emphasis on tumorigenesis, EMT and the self-renewal of CSCs. We observed marked differences in IL-32θ expression levels between tumor and non-tumor regions of samples from colon cancer patients. Therefore, we sought to determine any correlation between IL-32θ expression levels and the progression of colon cancer. We hypothesized that downregulation of IL-32θ expression might contribute to the progression of tumors; and IL-32θ levels could be associated with the prognosis of a colon cancer patient. We postulated that IL-32θ could be applied to the treatment of various tumors.

## RESULTS

### IL-32θ expression levels correlate with the progression of human colon cancer

A comparison of the gene sequences encoding IL-32β and IL-32θ revealed that exon 6 was absent in IL-32θ [24]. Therefore, immunohistochemistry was not possible because IL-32θ-specific antibodies are not yet available. However, we were able to conduct reverse transcription polymerase chain reaction (RT-PCR) assays to detect and differentiate IL-32θ mRNAs from other IL-32 isoforms, in tissue samples (*n* = 85) from colon cancer patients (Supplementary Figure 1). We found that IL-32 was expressed in tumor and non-tumor regions, as previously reported [25]. The clinicopathological features of tumors have been summarized in Table 1. The expression levels of IL-32θ in non-tumor and tumor sections were lower than those for other isoforms of IL-32 (Supplementary Figure 1). We focused on IL-32θ expression levels in tumor regions and categorized these in Table 1. To determine any association between IL-32θ and tumor characteristics, we applied clinicopathologic parameters in the analysis of our results. We determined that IL-32θ expression levels were not associated with age; however, there was some correlation with gross type (*p* = 0.038), pathological stage of tumors (*p* = 0.048) and recurrence (*p* = 0.041). Our results indicate an association between IL-32θ levels and the progression of colon cancer.

**Table 1:** Clinicopathological correlation between IL-32θ expression with colon cancer patient

IL-32θ expression				
	*n*	Positive (%)	Negative (%)	*p* Value
**Total**	85	7 (10)	78 (90)	
**Age**				0.430
**> 60**	51	3 (22)	48 (78)	
**< 60**	34	4 (26)	30 (74)	
**Gender**				0.230
**Male**	50	6 (15)	43 (85)	
**Female**	35	1 (3)	34 (97)	
**Location**				0.592
**A**	9	1 (11)	8 (89)	
**D**	2	0 (0)	2 (100)	
**R**	39	5 (17)	34 (83)	
**S**	28	1 (4)	27 (96)	
**Other**	7	0 (0)	7 (100)	
**Gross type**				**0.038**
**Localized**	53	7 (15)	41 (85)	
**Infiltrative**	32	0 (0)	32 (100)	
**Size**				0.700
**< 5 cm**	33	2 (6)	31 (94)	
**> 5 cm**	52	5 (13)	47 (87)	
**Depth of invasion (pT)**				0.520
**T1**	2	0 (0)	2 (100)	
**T2**	10	2 (20)	8 (80)	
**T3**	72	5 (9)	67 (91)	
**T4**	1	0 (0)	1 (100)	
**Serosal invasion**				0.256
**N (T1, T2)**	12	2 (17)	10 (83)	
**Y (T3, T4)**	73	5 (9)	68 (91)	
**Pathological stage (pStage)**				0.104
**1**	8	0 (0)	8 (100)	
**2**	47	7 (10)	40 (90)	
**3**	26	0 (0)	26 (100)	
**4**	4	0 (0)	4 (100)	
**1, 2**	55	7 (16)	48 (84)	**0.048**
**3, 4**	30	0 (0)	30 (100)	
**Recur**				**0.041**
**N**	66	3 (5)	63 (95)	
**Y**	19	4 (29)	15 (71)	
**Metastasis**				0.650
**N**	66	5 (8)	61 (92)	
**Y**	19	2 (19)	17 (81)	

Boldface indicates statistical significance.

**Figure 1:**
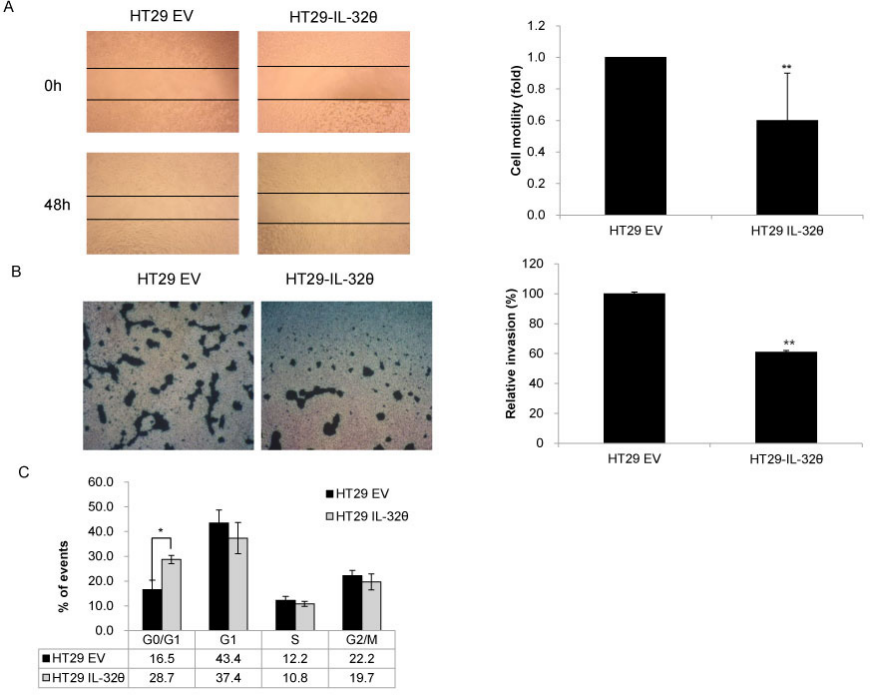
IL-32θ inhibits colon cancer tumorigenicity *in vitro* (**A**) The migration abilities of HT29 and HT29-IL-32θ cells were analyzed by wound healing assays. Wounds were made using pipette tips and cell motility was observed after 48 h. (**B**) The invasive capacities of HT29 and HT29-IL-32θ cells were analyzed using matrigel invasion assays. Phase-contrast microscopy images are shown. Values are presented as the mean ± S.D. (*N* = 3). (**C**) DNA content of HT29 and HT29-IL-32θ cells as determined by flow cytometry. *p < 0.05 and **p < 0.005.

### IL-32θ inhibits the proliferation of colon cancer cells *in vitro*

We generated a colon cancer cell line that stably expressed IL-32θ (HT29-IL-32θ) and evaluated the effects of this IL-32 isoform on the proliferation of cells. Cell proliferation was assessed using migration and invasion assays. For wound healing assays, phase-contrast microscopy images of control HT29 and HT29-IL-32θ cell cultures were acquired at 0 and 48 h after wounding. Overexpression of IL-32θ significantly reduced cell motility (Figure 1A) and markedly decreased the invasiveness of HT29 cells (Figure 1B). Our findings indicated that IL-32θ can inhibit the migration and invasion of colon cancer cells *in vitro*. We also examined whether IL-32θ can inhibit the cell cycle in HT29 cells using flow cytometry. We found that IL-32θ arrested the cell cycle, in the G0/G1 phase, for 16.5 and 28.7% of HT29 and HT29-IL-32θ cells, respectively (Figure 1C). These observations were consistent with the notion that IL-32θ might possess the ability to inhibit the growth and metastasis of tumors.

### IL-32θ inhibits the properties of colon CSCs

**Figure 2:**
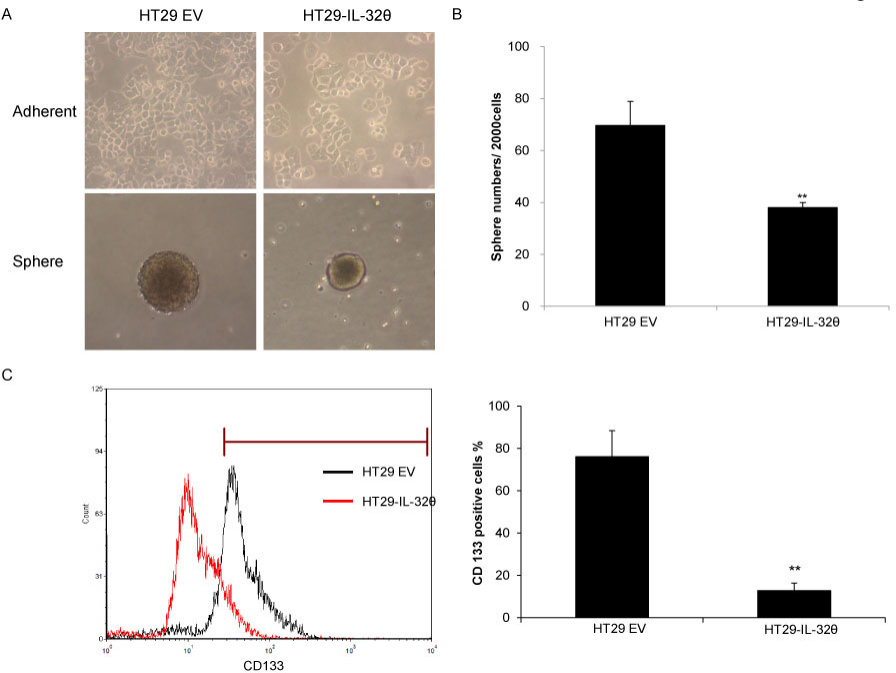
IL-32θ inhibits various properties of cancer stem cells (CSCs) (**A**) Representative images of parent cells and spheroid bodies for HT29 and HT29-IL-32θ cultures. (**B**) Spheroid bodies of HT29 and HT29-IL-32θ cells with a diameter greater than 50 μm were assessed after 7 days. (**C**) There were significantly less CD133^+^ cells in HT29-IL-32θ cultures compared with those in HT29 cultures.

We examined the CSC properties of HT29 and HT29-IL-32θ cells by culturing them in sphere-inducing media. We generated floating spheroid bodies to assess the *in vitro* self-renewal and differentiation capacities of these cells [26]. IL-32θ failed to alter the morphology of adherent cells; however, the sizes of spheroid bodies were reduced (Figure 2A). Similar results were seen with the HCT116 colon cancer cell line (Supplementary Figure 2). In addition, there were fewer spheres with a diameter greater than 50 μm that formed in HT29-IL-32θ cultures than HT29 cultures (Figure 2B). Our findings suggest that IL-32θ inhibits CSC properties of colon cancer cells. We quantified the number of CD133^+^ cells that were present in HT29 and HT29-IL-32θ cultures. There were less CD133^+^ cells in the HT29-IL-32θ cultures than in the HT29 cultures (Figure 2C), further indicating that IL-32θ inhibits colon CSC properties.

**Figure 3:**
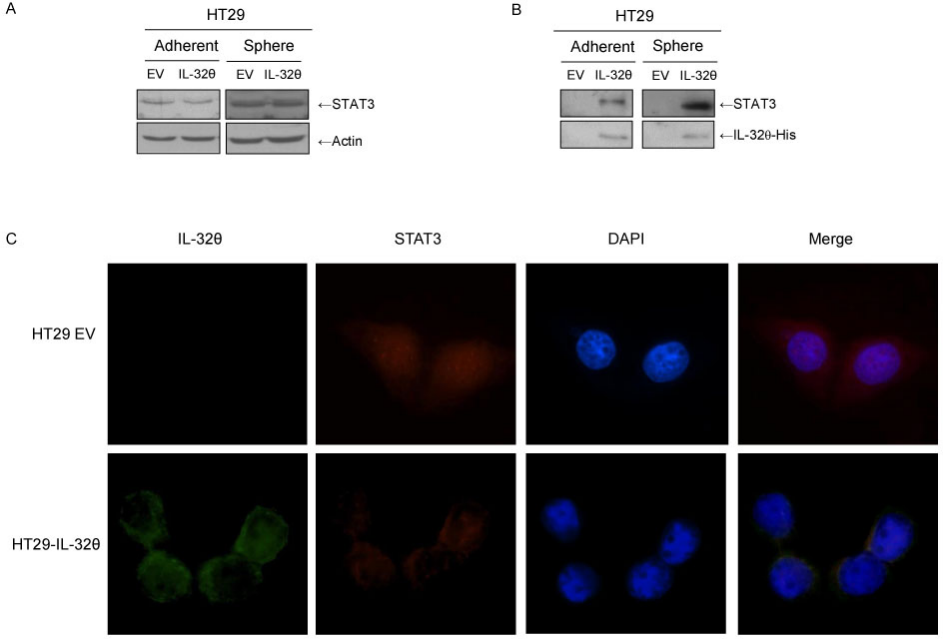
IL-32θ inhibits nuclear translocation of STAT3 through direct binding in HT29 cells (**A**) STAT3 expression levels in normal HT29 cells, HT29 CSCs and HT29-IL-32θ cells. Actin was used as an internal control. (**B**) Pull-down assays were conducted with IL-32θ-His. (**C**) STAT3 (Red) and IL-32θ (Green) were visualized by immunofluorescent staining (1000 × magnification). To visualize the nuclei of cells, DAPI was used.

Results from recent studies suggest that STAT3 is involved in colon CSC signaling [27]. We have also previously shown that IL-32θ can inhibit STAT3 *via* PKCδ [24]. We investigated STAT3 expression levels and observed that IL-32θ did not alter expression levels of this transcription factor (Figure 3A). To verify direct binding between IL-32θ and STAT3, we performed pulldown assays using a recombinant hexa-histidine-tagged IL-32θ protein (IL-32θ-His) purified from *Escherichia coli*. Exogenous IL-32θ-His was used to capture the IL-32θ binding molecules. IL-32θ directly binds with STAT3 in adherent HT29 cultures and in HT29 cultures containing spheroid bodies (Figure 3B). Confocal microscopy revealed that IL-32θ inhibits the nuclear translocation of STAT3, suggesting that IL-32θ inhibits STAT3 transcriptional activity (Figure 3C). In the absence of IL-32θ, STAT3 was found in both the nucleus and the cytosol. In the presence of IL-32θ, STAT3 remained in the cytosol. The inhibition of STAT3 nuclear translocation by IL-32θ through direct binding led to suppressed transcription of downstream factors.

**Figure 4:**
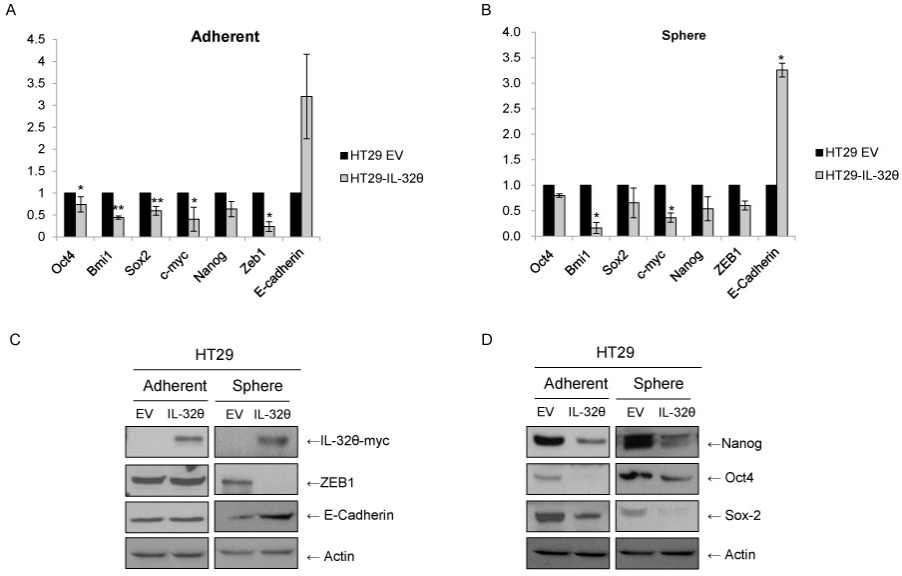
IL-32θ inhibits stemness and epithelial-mesenchymal transition (EMT) of HT29 cells (**A**) qPCR assays were used to determine the expression levels of stemness genes and EMT markers. The mRNA expression levels were normalized to those for *B2M*. Values are presented as the mean ± S.E.M (*N* = 5). (**B**) Western blotting analysis of stemness and EMT markers, with actin used as a loading control. *p < 0.05 and **p < 0.005).

STAT3 can induce EMT of colon cancer cells [15], activate ZEB1 and suppress E-cadherin [28]. Therefore, we used qPCR assays (Figure 4A and 4B) and western immunoblotting (Figure 4C) to determine ZEB1 and E-cadherin levels. *ZEB1* expression was downregulated in spheroid bodies but not in adherent cells. *E-cadherin* expression levels were higher in HT29-IL-32θ than in HT29 spheroid bodies as expected.

We investigated whether IL-32θ overexpression inhibits the stemness of CSCs. Expression levels of stemness factors, such as Oct4, Bmi1, Sox2, c-myc and Nanog, were determined by qPCR (Figure 4A and 4B) and western blotting (Figure 4D). Analysis using qPCR assays showed that IL-32θ overexpression resulted in a significant decrease in the expression levels of stemness genes. The similar expression patterns of *Nanog*, *Oct4* and *Sox2* in HT29-IL-32θ cells indicate that IL-32θ can suppress the self-renewal properties of CSCs. These molecular events, consistent with the reduction of sphere formation (Figure 2A), clearly indicate that IL-32θ is a negative regulator of stemness in colon CSCs. We showed that EMT and stemness of colon cancer cells might be inhibited by IL-32θ, implying that IL-32θ could be used to eliminate colon CSCs.

### IL-32θ suppresses the growth, self-renewal and EMT-associated properties of xenograft tumors

**Figure 5:**
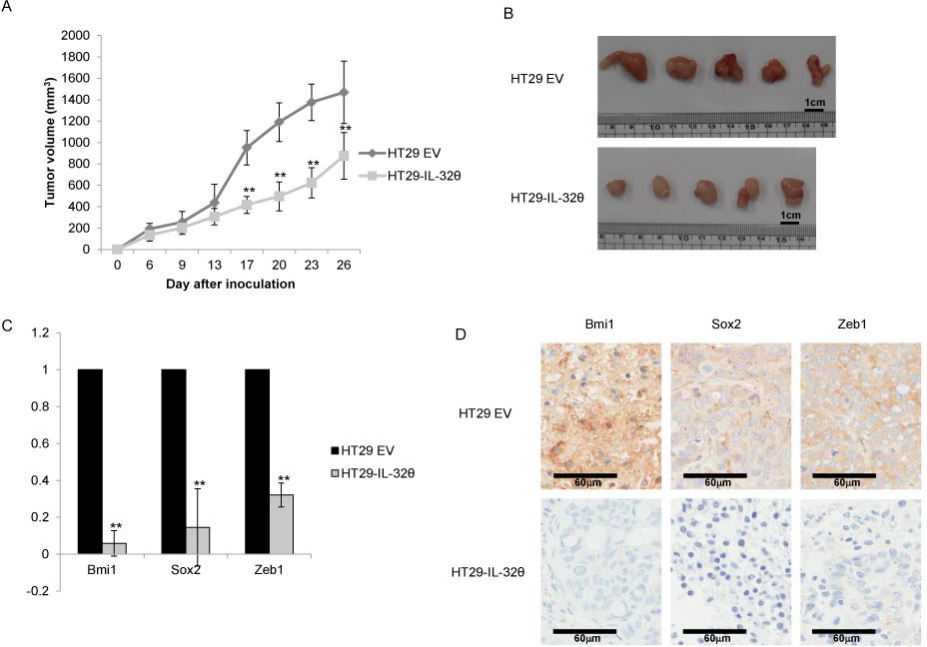
IL-32θ inhibits the tumorigenic ability of HT-29 cells *in vivo* (**A**) The overexpression of IL-32θ inhibits the growth of colon cancer xenografts. (**B**) Colon tumors in xenografts. Tumors from mice injected with normal HT-29 cells (Top) or HT-29-IL-32θ cells (Bottom) are shown. Tumors were collected after 26 days. (**C**) The effects of IL-32θ on tumor tissues were determined by qPCR. The expression levels of mRNAs were normalized to those for B2M. The values presented are relative to those for cells transfected with empty vector. (**D**) Representative immunohistochemical micrographs (400 × magnification) showing *Bmi1, Sox2* and *Zeb1* in tumor tissues. Data are presented as the mean ± S.D. **P < 0.005.

Athymic nude mice were intraperitoneally injected with HT29 or HT29-IL-32θ cells. Tumor formation was reduced in mice administered HT29-IL-32θ cells compared with those given HT29 cells (Figure 5A and 5B). In HT29-IL-32θ cells, expression levels of Bmi1, Sox2 and ZEB1 transcripts were lower than those in HT29 cells (Figure 5C). Immunohistochemical staining revealed that *Bmi1, Sox2* and *ZEB1* were downregulated to a greater extent in HT29-IL-32θ cells than in HT29 cells (Figure 5D).

## DISCUSSION

Cytokines secreted by either cancer cells or surrounding stromal cells can induce tumorigenesis [29]. Results from recent studies have revealed that the inflammatory microenvironment is able to promote oncogenesis. IL-1β promotes the self-renewal and oncogenic ability of gliomas [30], while IL-6 and IL-8 activate oncogenic ability of breast [31] and colon [13] CSCs. IL-32 and its isoforms are correlated in various diseases. Its aberrant production is linked to oncogenesis, the progression of multiple types of cancer and the suppression of tumors [25, 32, 33]. As an example, IL-32γ enhances TNF-α-induced cell death in cases of colon cancer [34] and inhibits the growth of cancer cells by blocking the NF-κB and STAT3 pathways [35]. Similar with this observations, we also showed that IL-32γ inhibited the formation of spheroid bodies, EMT and the transcription of stemness factors (Supplementary Figure 3) [34]. But to date, the exact mechanism by which IL-32 suppresses tumor growth remains unclear. The new isoform of IL-32 that we discovered, IL-32θ, appears to suppress cancer progression. The expression of IL-32θ negatively correlated with disease stage, where high-grade tumors exhibit lower levels of IL-32θ mRNAs than those in low-grade tumors (Table 1). Our findings imply that IL-32θ expression occurs at relatively early stages of tumor formation and disappears as the disease progresses. Therefore, we focused on the role of IL-32θ in colon cancer. We found that IL-32θ overexpressing cells displayed decreased invasiveness and oncogenic capabilities and exhibited a decreased ability to form spheroid bodies. Our results provide evidence that IL-32θ likely inhibits the progression of colon cancer and its recurrence, through the regulation of self-renewal and EMT.

**Figure 6:**
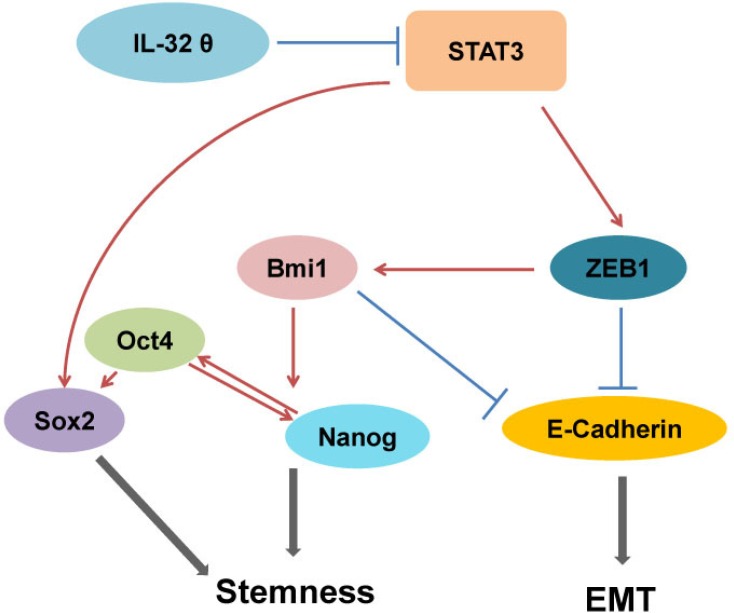
Inhibition of stemness and EMT by IL-32θ The stemness of HT29 cells and EMT were inhibited by IL-32θ *via* inhibition of the STAT3-ZEB1 pathway and subsequent regulation of downstream factors.

Although the signaling pathway for IL-32θ in colon cancer has yet to be revealed, it has been shown that IL-32θ inhibits the transcriptional activity of STAT3 [24]. STAT3 has been shown to induce the tumorigenesis of human colorectal cancer; therefore we assumed that IL-32θ inhibits the activity of STAT3, tumorigenesis and various properties of CSCs. In addition, we postulate that IL-32θ inhibits EMT and the stemness of CSCs *via* the inhibition of STAT3 pathways. IL-32θ directly binds to STAT3 and inhibits its nuclear translocation. This subsequently results in downregulation of ZEB1, Sox2 and Bmi1 (Figure 6).

The IL-32θ-induced inhibition of STAT3 results in the downregulation of ZEB1, Bmi1 and also upregulation of E-cadherin signaling in colon cancer cells. STAT3 indirectly inhibits E-cadherin, which is involved in EMT, given that a loss of E-cadherin triggers tumor metastasis and EMT. Li *et al*. found that that ZEB1 is upregulated by IL-1β and has a role in the induction of Bmi1 [19]. It has been reported that Bmi1 induces self-renewal and oncogenic potential of colon CSCs. CD133^+^ cells can also express other markers that are indicative of stem cells [36]. Of the CSC markers we assessed, we found that Bmi1 expression was reduced by IL-32θ. We also found that IL-32θ decreased Bmi1 mRNA levels in CD133^+^ cells (Supplementary Figure 4). We believe that the reduced ability for self-renewal of IL-32θ overexpressing cells might be due to decreased levels of Bmi1 expression. Based on our *in vitro* and *in vivo* data, we concluded that the upregulation of IL-32θ suppressed EMT and stemness of colon CSCs. Ectopic IL-32θ inhibited the STAT3-ZEB1 pathway and could possibly be used for colon CSC therapy. In conclusion, our result reveal that a new isoform of IL-32, IL-32θ, inhibits CSC self-renewal and EMT, which is relevant to the growth of tumors and the recurrence of colon cancer.

## MATERIALS AND METHODS

### Patient samples

Frozen colon cancer tissue samples were obtained from 85 patients that had undergone curative surgical resection and information on clinical data at Chungnam National University Hospital between 2009 and 2011. Human colon cancer samples were only collected from patients who had given informed consent.

### Cell culture and spheroid body formation

HT29 and HCT116 (ATCC, Manassas, VA, USA) cell lines were cultured in Dulbecco's modified Eagle's medium (Hyclone, Logan, UT, USA) supplemented with 10% heat-inactivated fetal bovine serum (FBS; Hyclone) at 37°C/5% CO_2_. To generate spheroid bodies, 1,000 cells/mL were seeded on ultra-low attachment pates (Corning, Lowell, MA, USA) and incubated at 37°C/5% CO_2_. Cells were cultured in DMEM/F12 (Gibco, Carlsbad, CA, USA) with B-27 supplement (Gibco), 10% FBS, basic fibroblast growth factor (Sigma-Aldrich, Poole, UK) and epidermal growth factor (Calbiochem, San Diego, CA, USA) for 7 days at 37°C/5% CO_2_.

### Generation of an IL-32θ-overexpressing cell line

We transfected HT29 cells with the pcDNA3.1 (+)-6× Myc-IL-32θ vector, as described previously [24]. Cells were seeded into 6-well plates (1 × 10^5^ cells/well) and transfected with 3 μg of pcDNA3.1–6× myc-IL-32θ or pcDNA3.1(+)-6× myc, using Lipofectamine^®^ 2000 (Invitrogen, Carlsbad, CA, USA) according to the manufacturer's instructions. After 48 h, cells were trypsinized and incubated in medium containing 600 μg/mL neomycin for 2 weeks. Neomycin-resistant colonies were then pooled and expanded.

### Migration assay

To determine cell motility, HT29 and HT29-IL-32θ cells (1 × 10^5^ cells/well) were seeded in 24-well plates and incubated at 37°C/5% CO_2_ overnight. Wounds were made using 200-μL pipette tips. Cells were then washed twice with Hank's balanced salt solution to remove floating cells and fresh medium was added. Cells migrating from the edge of the wound were photographed from five random fields of view, at 0 and 48 h after wounding.

### Invasion assay

Invasion assays were performed as described previously [37]. Briefly, 8-μm upper chambers (Millipore, Billerica, MA, USA) were coated with ECM gel (Sigma-Aldrich, Poole, UK) and then HT29 and HT29-IL-32θ cells (1 × 10^5^ cells/well) in serum-free media were seeded in these chambers. Complete medium was added to the lower chambers as a chemoattractant to induce invasion. After 24 h, membranes were fixed, stained and enumerated. Images were acquired from three random fields of view.

### Isolation of CD133^+^ cells

An antibody against CD133 was purchased from Miltenyi Biotec (Bergisch gladbach, Germany). Briefly, HT29 and HT29-IL-32θ cells were cultured as spheroid bodies. CSCs were dissociated with Accutase^®^ (Sigma) and stained with the CD133 antibody at 37°C for 15 min. Populations of CD133^+^ cells were sorted by flow cytometry.

### Cell cycle analysis

To analyze the cell cycle of HT29-IL-32θ cells, they were trypsinized and then fixed with 70% ethanol. Cells were stained with propidium iodide (BD Biosciences, Franklin Lakes, NJ, USA) for 30 min and then up to 10,000 events were assessed with a BD flow cytometer (BD Biosciences). The DNA contents of cells were analyzed using CellQuest software (BD Biosciences).

### Reverse transcription polymerase chain reaction analysis

Total RNA was prepared from HT29 and HT29-IL-32θ adherent cells and CSCs using TRIzol (Molecular Research Center, Cincinnati, OH, USA). We synthesized cDNA from total RNA samples using a first-strand cDNA synthesis kit (Fermentas, Burlington, Ontario, Canada). Real-time qPCR assays were performed using a relative quantification protocol, the Exicycler™ 96 Real-Time Quantitative Thermal Block (Bioneer, Daejeon, Korea) and SYBR Premix Ex Taq (Takara, Otsu, Japan). We used oligonucleotide primer sequences that were specific for *Oct4* (5′-GGT TCT CGA TAC TGG TTC GC-3′ and 5′-GTG GAG GAA GCT GAC AAC AA-3′), *Bmi1* (5′-AAA TGC TGG AGA ACT GCA AAG-3′ and 5′-CTG TGG ATG AGG AGA CTG C-3′), *Sox2* (5′-GCT TAG CCT CGT CGA TGA AC-3′ and 5′-AAC CCC AAG ATG CAC AAC TC-3′), *c-myc* (5′-GGC CTT TTC ATT GTT TTC CAA CT-3′ and 5′-GGA ACG AGC TAA AAC CCA GCT-3′), *Nanog* (5′-ATG GAG GAG GGA AGA GGA GA-3′ and 5′-GAT TTG TGG GCC TGA AGA AA-3′), *ZEB1* (5′-GCC AAT AAG CAA ACG ATT CTG-3′ and 5′-TTT GGC TGG ATC ACT TTC AAG-3′), E-cadherin (5′-GAA GGT GAC AGA GCC TCT GGA T-3′ and 5′-ATC GGT TAC CGT GAT CAA AAT C-3′) and *B2M* (5′-TCT CTG CTG GAT GAC GTG AG-3′ and 5′-TAG CTG TGC TCG CGC TAC T-3′).

### Western blotting analysis

Cells were harvested and lysed with buffer containing 20 mM HEPES (pH 7.5), 150 mM NaCl, 10% glycerol, 20 mM β-glycerophosphate, 1% Triton X-100, 1 mM EDTA and 2 mM EGTA. Lysates were boiled at 100°C for 5 min. Equal amounts of total proteins were resolved using 10–12% sodium dodecyl sulfate polyacrylamide gel electrophoresis (SDS-PAGE). Proteins were transferred to nitrocellulose membranes (Millipore) and incubated with antibodies against STAT3, ZEB1, Actin (Santa Cruz Biotechnology), E-cadherin, Oct4 (Abcam, Cambridge, MA, USA), Sox2, Nanog (Cell signaling) and Myc (Millipore) overnight at 4°C. Membranes were then washed with Tris-buffered saline containing 0.05% Tween 20 and incubated with mouse, rabbit (Thermo Scientific), or goat (Santa Cruz Biotechnology) horseradish peroxidase-conjugated secondary antibodies for 1 h at room temperature. Specific protein bands were detected using SuperSignal Chemiluminescent Substrate (Pierce, Rockford, IL, USA), according to the manufacturer's instructions.

### Immunofluorescence

The procedure used for immunocytochemistry has been described previously [24]. Briefly, cells were seeded on glass coverslips, incubated at 37°C/5% CO_2_ overnight and then transfected with pcDNA3.1(+)-6× Myc-IL-32θ. After 24 h, cells were permeabilized and fixed with 100% acetone and blocked with 0.1% (w/v) bovine serum albumin (BSA). Antibodies against myc and STAT3 were diluted 1:100 in 0.1% (w/v) BSA and incubated overnight at room temperature. Secondary mouse and rabbit antibodies (Millipore) were diluted 1:200 in 0.1% (w/v) BSA and incubated for 1 h at room temperature. Coverslips were mounted with Vectashield containing 4′, 6-diamidino-2-phenylindole (Vector Laboratories, Burlingame, CA, USA) and cells were visualized by confocal microcopy (Carl Zeiss, Oberkochen, Germany).

### Pull-down assays

To generate the IL-32θ expression vector, we used the pPROEX HTa and pcDNA3.1(+)-6× Myc-IL-32θ vectors. Both vectors were digested with *Eco*RI and *Xho*I for 1 h and then ligated with T4 ligase for 1 h. The sequence of the resulting plasmid was verified by sequencing and transformed into *E. coli* BL21-RIL competent cells. Recombinant colony were picked and incubated overnight at 37°C in Luria-Bertani broth. Once the optical density of cultures at 540 nm was 0.6–0.8, we added 0.1 mM isopropyl β-D-1-thiogalactopyranoside to induce protein expression. Cultures were incubated for 4 h at 30°C and then sonicated to lyse cells. Proteins were purified from lysates by column chromatography and then dialyzed. The IL-32θ-His protein was used as the prey protein. HT29 cell lysates containing endogenous STAT3 were incubated with purified IL-32θ-His overnight. Samples were then washed three times with radioimmunoprecipitation assay buffer, eluted and then subjected to SDS-PAGE.

### Xenografts

The *ex vivo* tumorigenic abilities of HT29 and HT29-IL-32θ cells were determined by xenograft tumor formation assays. We injected 2 × 10^5^ cells with matrigel (Sigma) subcutaneously into both flanks of 5-week-old female athymic nude mice from Orient Bio (Sungnam, Korea). The length and width of tumors were measured using calipers every 5 days. After 26 days, tumors were harvested from euthanized mice. The acquired tumor tissues were fixed with 10% neutral-buffered formalin, embedded in paraffin and sectioned (3-μm thickness) for use in immunohistochemical analyses. All animal procedures were conducted according to the guidelines of the Institutional Animal Care and Use Committee of the Korea Research Institute of Bioscience and Biotechnology.

### Immunohistochemistry

Formalin-fixed paraffin embedded tumor tissue sections were immersed in citrate buffer and boiled for 15 min in a pressure cooker to retrieve antigens. Sections were washed for 5 min and endogenous peroxidase activity was blocked with 3% (v/v) H_2_O_2_ for 10 min. Non-specific binding sites were blocked with Protein Block (Dako, Carpinteria, CA, USA) for 20 min. Sections were incubated with the appropriate primary antibody overnight and then the appropriate secondary antibody for 30 min. Diaminobenzidine tetrahydrochloride was used as substrate, as sections were then counterstained with hematoxylin.

### Statistical analyses

Fisher's exact test was used for comparisons between two groups. Statistically significant differences were determined by Student's *t*-test. Data are expressed as means ± S.E.M. A *p*-value less than 0.05 was considered significant, while a *p*-value less than 0.005 was considered very significant.

## SUPPLEMENTARY MATERIALS FIGURES



## Abbreviations

EMT: Epithelial-mesenchymal transition
CSCs: cancer stem cells
IL: Interleukin
STAT3: signal transducer and activator of transcription 3
RT-PCR: reverse transcription polymerase chain reaction
qPCR: quantitative PCR
SDS-PAGE: sodium dodecyl sulfate polyacrylamide gel electrophoresis
BSA: bovine serum albumin
FBS: fetal bovine serum
