# Supplementary material for: IL-32θ inhibits stemness and epithelial-mesenchymal transition of cancer stem cells via the STAT3 pathway in colon cancer
Source: Oncotarget. 2016 Jan 25;7(6):7307–17. doi: 10.18632/oncotarget.7007 (PMC4872787; doi:10.18632/oncotarget.7007)
Supplement: Supplementary file 1 [file oncotarget-07-7307-s001.pdf]

# IL-32θ inhibits stemness and epithelial-mesenchymal transition of cancer stem cells *via* the STAT3 pathway in colon cancer

## Supplementary Materials

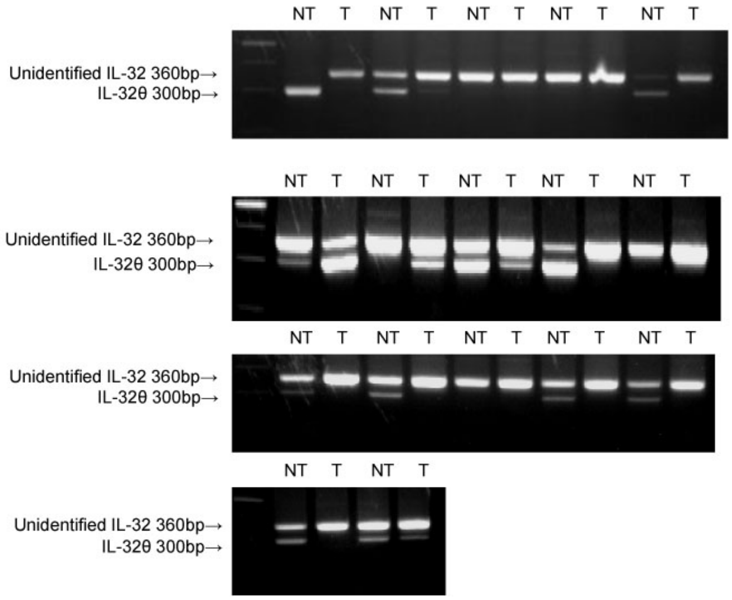


**Supplementary Figure S1: IL-32θ downregulation is correlated with the tumor malignancy in colon cancer.** RT-PCR was performed to examine IL-32θ expression levels in non tumor, tumor region of patient tissues. IL-32θ doesn't have exon 6 compared to IL-32β, resulted in size difference (IL-32θ:300 bp; other IL-32 isoform:360 bp).

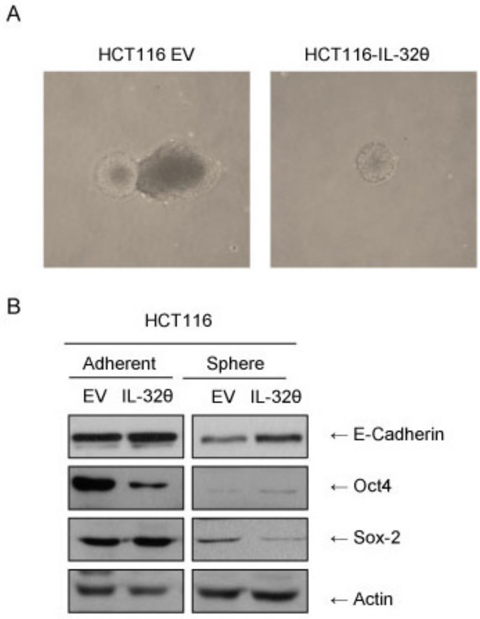


**Supplementary Figure S2: IL-32θ regulates sternness and epithelial-mesenchymal transition related factor in HCT116 cells.** (**A**) Representative images of sphere cell in HCT116 and HCT116-IL-32θ cells. HCT116 and HCT116-IL-32θ cells were seeded in sphere media (2000 cells/well) for sphere formation. (**B**) Western blots showing the expression of sternness and EMT markers. Actin was used as a loading control.

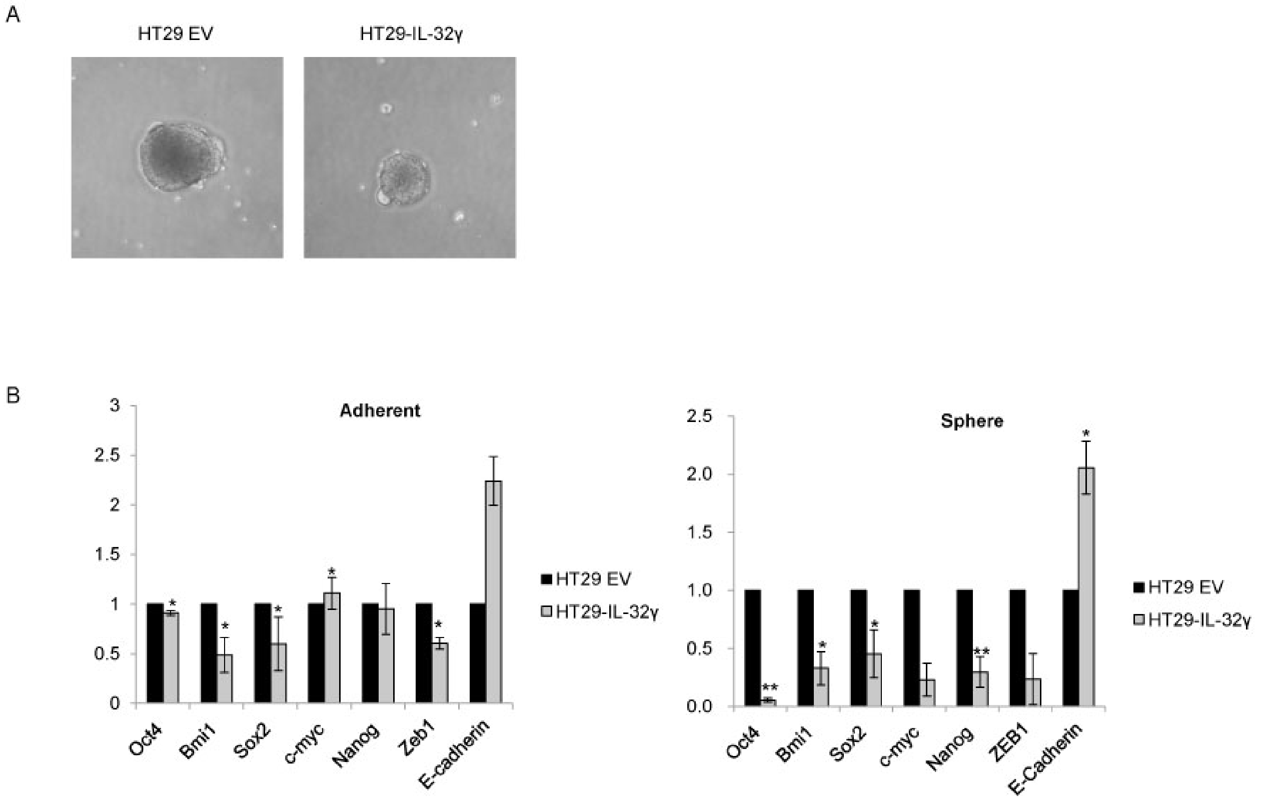


**Supplementary Figure S3: IL-32$\gamma$ regulates sternness and epithelial-mesenchymal transition related factor in HT29 cells.** (**A**) Representative images of sphere cell in HT29 and HT29-IL-32y cells. HT29 and HT29-IL-32$\gamma$ cells were seeded in sphere media (2000 cells/well) for sphere formation. (**B**) Quantitative real time PCR data of sternness and EMT markers. RNA levels were normalized to B2M. Data means + S.E.M ($n = 5$). *P*-values were determined by Student's *t*-test (* < 0.05, ** < 0.005).

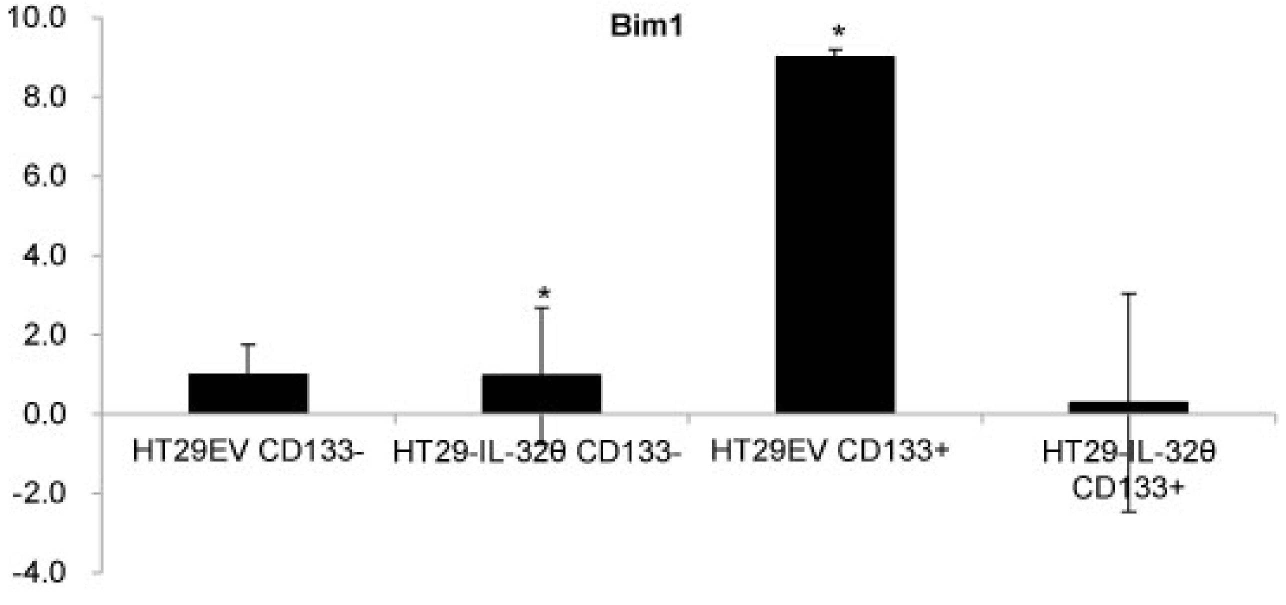


**Supplementary Figure S4: CD133$^+$ and CD133$^-$ cells were seeded in sphere media for second generation.** After 7 days, cells were harvested and analyzed by qPCR. Values denote the mean ± SEM of three independent assays. Statistical significance was determined by Student's *t*-test (*$p < 0.05$).
